# Singlet Oxygen, Photodynamic Therapy, and Mechanisms of Cancer Cell Death

**DOI:** 10.1155/2022/7211485

**Published:** 2022-06-25

**Authors:** Prabal Singh Maharjan, Hitesh Kumar Bhattarai

**Affiliations:** Department of Biotechnology, Kathmandu University, Dhulikhel, Nepal

## Abstract

Photodynamic therapy (PDT) can be developed into an important arsenal against cancer; it is a minimally invasive therapy, which is used in the treatment or/and palliation of a variety of cancers and benign diseases. The removal of cancerous tissue is achieved with the use of photosensitizer and a light source, which excites the photosensitizer. This excitation causes the photosensitizer to generate singlet oxygen and other reactive oxygen species. PDT has been used in several types of cancers including nonmelanoma skin cancer, bladder cancer, esophageal cancer, head and neck cancer, and non-small cell lung cancer (NSCLC). Although it is routinely used in nonmelanoma skin cancer, it has not been widely adopted in other solid cancers due to a lack of clinical data showing the superiority of PDT over other forms of treatment. Singlet oxygen used in PDT can alter the activity of the catalase, which induces immunomodulation through HOCl signaling. The singlet oxygen can induce apoptosis through both the extrinsic and intrinsic pathways. The extrinsic pathway of apoptosis starts with the activation of the Fas receptor by singlet oxygen that leads to activation of the caspase-7 and caspase-3. In the case of the intrinsic pathway, disruption caused by singlet oxygen in the mitochondria membrane leads to the release of cytochrome c, which binds with APAF-1 and procaspase-9, forming a complex, which activates caspase-3. Mechanisms of PDT action can vary according to organelles affected. In the plasma membrane, membrane disruption is caused by the oxidative stress leading to the intake of calcium ions, which causes swelling and rupture of cells due to excess intake of water, whereas disruption of lysosome causes the release of the cathepsins B and D, which cleave Bid into tBid, which changes the mitochondrial outer membrane permeability (MOMP). Oxidative stress causes misfolding of protein in the endoplasmic reticulum. When misfolding exceeds the threshold, it triggers unfolding protein response (UPR), which leads to activation of caspase-9 and caspase-3. Finally, the activation of p38 MAPK works as an alternative pathway for the induction of MOMP.

## 1. Introduction

**Figure 1:**
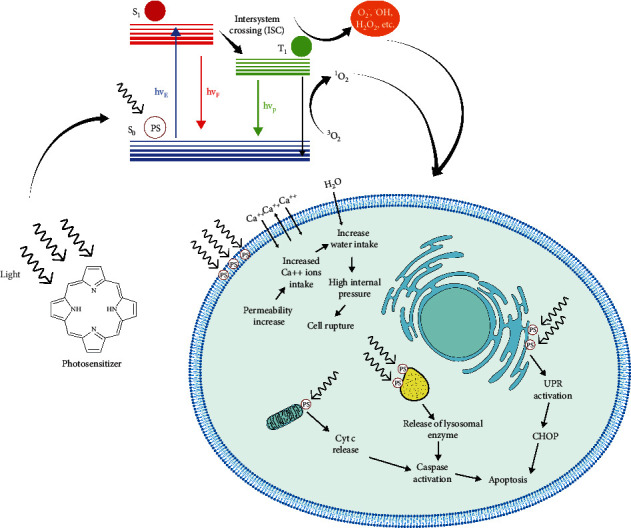
Diagrammatic summary of the paper.

Developing chemotherapy against cancers is particularly difficult. Cancer cell is, in many ways, like every other cell in the body, and drugs that kill cancer cells also kill normal cells. Early cancer chemotherapy consisted of poisons. Mustard gas was used in wars to kill humans. At right dosages, it could be used as an anticancer drug [1]. Similarly, a purine, 6-mercaptopurine, is highly toxic but can be used as a chemotherapeutic agent [2]. Early innovations in chemotherapy involved giving a concoction of drugs to patients to prevent relapse. The first experiment used 6-mercaptopurine and methotrexate to treat cancer [3]. As the effects of the poisons multiplied, the double dose was more toxic than the single dose. However, the combination regiment led to higher remission rates. The ultimate set of poisons used to try to cure cancer was a combination known as VAMP acronym for vincristine, amethopterin, mercaptopurine, and prednisone. The severely sick patients got sicker and came very close to death. The bone marrow of the patients was damaged. At the end of the tunnel, there was hope. When finally the bone marrow started recovering, cancer cells did not come back [4] (Figure 1).

Like many predecessor cancer drugs, singlet oxygen is also a poison [5]. It causes necrosis and apoptosis and leads to cancer death. The important advantage of using singlet oxygen as a cancer therapy is the ability to control the location of death so that only the cancer cells are targeted, and other cells are spared. In this review of the mechanism of singlet oxygen-induced cell death, we first introduce the concept of singlet oxygen.

### 1.1. Singlet Oxygen Introduction

**Figure 2:**
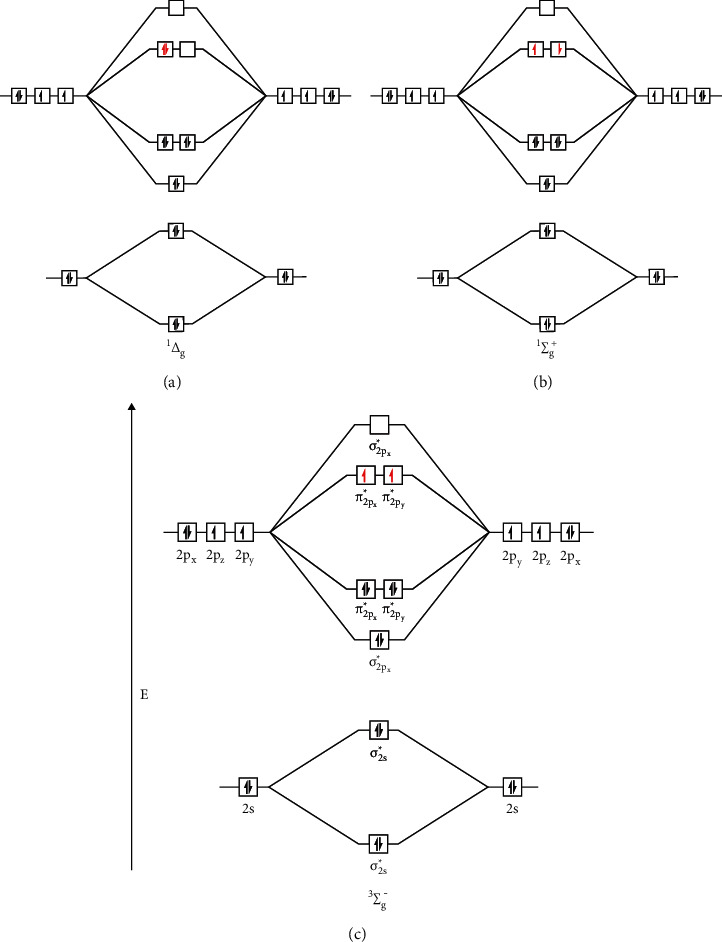
Molecular orbital diagram of two singlet excited states and a triplet state of oxygen. There are three different states of oxygen molecules, i.e., ^3^Σ_g_^−^, ^1^Δ_g_, and ^1^Σ_g_^+^. These states differ in the placement of two electrons in two antibonding orbitals (AOs). In the case of the triplet state (^3^Σ_g_^−^), the two electrons occupy two AOs singly and have the same spin, whereas in the first singlet state (^1^Δ_g_) the two electrons fill one AO (^1^Σ_g_^+^) without filling another, which puts the second state in the higher energy state. The first singlet state does not follow Hund's rule. The second singlet state, in which the two electrons occupy two AOs singly but do not have the same spin, also does not follow Hund's rule. The ground state oxygen molecule has one unpaired electron in each antibonding orbital and has like spins. When the ground state triplet oxygen (^3^Σ_g_^−^) (b) is excited by the transfer of energy, denoted by E, it changes to singlet oxygen (^1^Δ_g_) (a), the first excited state. ^1^Δ_g_ has paired electrons in only one antibonding orbital with opposite spin and is unstable and reactive. Even more unstable singlet oxygen (^1^Σ_g_^+^) (c), the second excited state, is formed by absorbing more energy where two electrons with opposite spin are aligned in two different antibonding orbitals. Usually, ^1^Δ_g_ is more stable in comparison with ^1^Σ_g_^+^, so the unstable form converts into a more stable ^1^Δ_g_.

Molecular oxygen is the second most abundant gas in the atmosphere and plays a critical role in the sustenance of life and the extinction and destruction of materials [6]. It is widely taught in high school textbooks that an oxygen atom has a total of 8 electrons, which means 2 electrons in 2s orbital and 2 electrons in 2p_*x*_ and 1 electron each in 2p_*y*_ and 2p_*z*_ orbitals [7]. When two oxygen atoms form orbitals, 2s orbitals combine to form bonding and antibonding sigma 2s orbitals, both of which are doubly occupied. p_*x*_, p_*y*,_ and p_*z*_ orbitals come to form one sigma bonding and one antibonding orbital and two pi sigma and anti-sigma orbitals. In the ground state of molecular oxygen, sigma and pi bonding orbitals are fully occupied. Two remaining electrons are found in two antibonding pi orbitals [8]. They both have the same spin. This is called the triplet oxygen ground state and is denoted as ^3^Σ_g_^−^ [6]. Due to electronic excitation, if an unpaired electron in the antibonding pi orbitals moves to another antibonding pi orbital and changes its spin, excited singlet oxygen species are formed. It is denoted as ^1^Δ_g_. Another excited state of oxygen occurs when the spin of one of the antibonding pi orbitals is flipped, which is the second excited state of singlet oxygen and is denoted as ^1^Σ_g_^+^ [9]. The number of electron spins possible gives the names singlet and triplet. The total quantum spin of singlet oxygen is 0 with only one possible arrangement of electron spins. On the other hand, the total quantum spin of triplet oxygen is 1 with three possible arrangements of electron spins [9] (Figure 2).

### 1.2. Photosensitized Production of Singlet Oxygen

**Figure 3:**
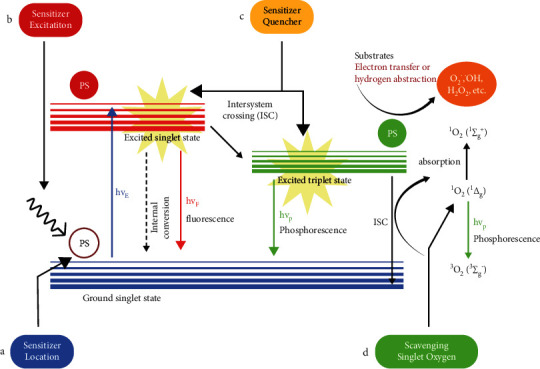
Jablonski diagram of photosensitizer excitation and production of singlet oxygen, and major nodes of improvement in photodynamic therapy. When the light excites the electron of a ground state photosensitizer, it gets promoted to an excited singlet state. The excited photosensitizer (PS) returns back to the ground state via two processes. It can directly lose energy through fluorescence (hvf) or get converted into a triplet state through the intersystem crossing. The triplet-state PS settles down to the ground state by transferring energy to oxygen molecules or transferring electrons or protons to substrates or via phosphorescence (hvp). Additionally, by absorbing more energy, singlet oxygen (^1^Δ_g_) (first excited state) can convert to singlet oxygen (^1^Σ_g_^+^) (second excited state). (a) Localized light focusing can be achieved using two-photo light, which is often in a longer wavelength range. It is not absorbed by the surrounding medium; only a photosensitizer that can sequentially absorb two photons of light will be excited. (b) Sensitizer location is very important in terms of effectiveness and the pathway it takes to cause cell death. For example, sensitizer activity in the mitochondria causes cell death through the intrinsic pathway, while sensitizer activity in the plasma membrane causes cell death due to cell rupture and extrinsic pathway. (c) Quenching agent (Q) can be fused with the sensitizer(s) to control the location of its excitation. Q can only be detached from the sensitizer under a certain cellular environment, which reduces the likelihood of sensitizer excitation in an unwanted location. (d) During the process of photodynamic therapy, singlet oxygen is not only produced in cancer cells but also produced in normal cells. The utilization of singlet oxygen scavengers in the surrounding cells can reduce the oxidative stress on normal cells.

Although singlet oxygen can also be produced from non-photochemical, preparative chemical methods, and phosphite ozonide, the photosensitized production of single oxygen is discussed in this study since this method is widely used to generate singlet oxygen in the treatment of cancers. Ground state oxygen is excited using wavelength usually in the visible range, using various sources such as LED and sunlight [6]. Photosensitizers are generally promoted to a higher energy state that can be a singlet state or a triplet state. Activation can be caused using a single photon or two photons. From the higher energy state, electrons reach lower energy states through internal conversion (IC) or to a different spin state through intersystem crossing (ISC). During the lowering of electrons of photosensitizer from the triplet state to a ground state, it can transfer energy to an oxygen molecule, which leads to simultaneous promotion of electrons in molecular oxygen from the ground state to the singlet state (Figure 3). There are two singlet oxygen states, ^1^Δ_g_ and ^1^Σ_g_^+^. ^1^Δ_g_ state is more stable, and in the rest of the study, the singlet state refers to this state of oxygen molecule. ^1^Δ_g_ can be further promoted to ^1^Σ_g_^+^, but immediate relaxation takes place [6]. Sunlight can promote these higher energy states; however, more controlled light sources (lasers and lamps) have been used in clinical treatment [10]. The light source in the clinic application has to have two properties: suitable spectral range and sufficient tissue penetration without scatter or loss [10]. An appropriate spectral range of the source radiation means that the sensitizer has to have the same absorption range [11], which is usually in the visible spectrum. If a red light is used, tissue penetration is low, so interstitial delivery of light has to be used to reach tumors that are deep lying. Generally, longer wavelength near-infrared (NIR) lights can have lower tissue scatter and can be used to penetrate deep-lying tumors [12]. Two-photon excitation is necessary for this kind of light [13]. Other than NIR light, X-rays can be used to penetrate deep tumors [14].

### 1.3. Properties and Measurement of Singlet Oxygen

What kind of effect the singlet oxygen will have will depend on the lifetime of the singlet oxygen and the distance it can travel within its lifetime. The diffusion coefficient of the medium will have an impact on these parameters of singlet oxygen [6]. Using earlier methods of calculations using indirect photo-bleaching experiments and extrapolating data from model solutions, lifetime of singlet oxygen was obtained as a minimum of 10–300 ns [15]. Later, accurate measurements gave the lifetime of singlet oxygen in the range of 15–30 *μ*s [16]. The diffusion radius of singlet oxygen can be estimated to be around 155 nm in neat water, whereas the value can be estimated to be around 550 nm in deuterated water [6]. Given that the typical eukaryotic cell diameter is about 10–30 *μ*m, singlet oxygen can be considered to be localized to organelles when considering its activity inside a eukaryotic cell. Thus, singlet oxygen is considered a selective and not a reactive intermediate [6].

Another important consideration is the measurement of singlet oxygen in the cell, which is an essential factor in understanding the effect of singlet oxygen in cells. For the measurement, the effect of the lifetime on the change in the solvent from water to deuterated water is used. The lifetime of singlet oxygen is known to be substantially longer in deuterated water (about 67 *μ*s), which is much longer than its lifetime in water, which is about 3.5 *μ*s [6]. The O-H vibrational mode is important in determining the lifetime of singlet oxygen, which leads to this difference in lifetimes. Replacement of water with deuterated water does not seem to have a detrimental effect on cells over a period of several hours. This time span is sufficient to observe several effects of singlet oxygen on cells. Another way to observe singlet oxygen in cells is directed by the spectral profile of single phosphorescence. Singlet oxygen has unique phosphorescence at about 7850 cm^−1^ (about 1275 nm) [17]. Although this signal is solvent-dependent, it can still be observed under different conditions. The only drawback of this signal is that it is weak and often needs to be measured under deuterated water. Often another luminescent probe molecule is used to detect and amplify this signal. However, this option is not without problems. The last option to detect singlet oxygen is by adding molecules of known specificity to quench the singlet oxygen [6]. This approach is a variation of the first method of using relative concentrations of H_2_O and D_2_O. In this approach, care must be taken not to introduce unwanted effects due to the addition of the quencher.

### 1.4. Photodynamic Therapy

Since the early civilization of Egyptians and Indians, it has been known that light can be used to treat certain diseases such as psoriasis, vitiligo, and skin cancers [18]. Early forms of therapy used sunlight or other forms of light to treat the diseased tissues directly. With the discovery of laser light, it could be used to directly treat the tissue of concern [19]. However, this type of treatment has important drawbacks. Other tissues that contain some amount of chromophore are affected, and very high-intensity laser light is required to achieve efficacy. These can lead to safety and logistic issues. Hence, a need for a more direct light therapy was felt. Photodynamic therapy (PDT) achieves such a high level of precision through the use of photosensitizer that localizes to the site of the tumor and the use of light directly at the oncogenic site. In the clinic, photosynthesizing agents can be topically applied, injected intravenously or intraperitoneally, or can be consumed orally. Oral consumption is easier but raises questions about bioavailability differences due to pharmacokinetic and pharmacodynamic variables [20]. After a certain amount of time, the light of a fixed wavelength is shown on the tumorigenic area, where, hopefully, the photosensitizer has reached so that light-induced type I and type II reactions can be initiated. In PDT, reactive oxygen species are generated by a type I or type II reactions that lead to tissue destruction [10].

Photodynamic therapy can be divided into two types based on the ROS generation mechanisms, i.e., type I PDT and type II PDT [21]. In the case of type I PDT, when the photosensitizer (PS) is under irradiation, the ground state type I PS absorbs the energy and converts it to the singlet state. The singlet PSs go back to their ground state via fluorescence emission or nonradioactive decay. On the other hand, they can get de-excited into the long-living triplet state through intersystem crossing (ISC). Thus, formed triplet-state PS can transfer electrons or the proton to substrates, i.e., electron-rich molecules or cell membranes [22]. The radicals thus generated are short-lived and highly reactive. They interact with water and oxygen molecules to produce hydrogen peroxide, superoxide anion, and hydroxyl radical [21], which cause specific damage to biomolecules and initiate a chain of radical reactions [23]. However, in type II PDT, the energy is transferred from the triplet PS to triplet oxygen (3O_2_), which produces cytotoxic singlet oxygen that specifically interacts with components of cells such as cell membrane, nucleus, mitochondria, endoplasmic reticulum, and lysosomes and causes cell death. Depending on the concentration and type of PS used in the reaction, both processes can co-occur [22]. Type I reaction often leads to more severe damage. In type I PDT, PSs are consumed and need to be regenerated [23].

While conventional type II PDT has immense anticancer potential, hypoxia severely hinders its efficiency. There has been considerable development in the new PDT paradigms, which could help us cope with the problem, such as fractional PDT, type I PDT, remote-controlled release of ^1^O_2_, and multimodal therapy [24]. In the context of type I PDT, the exact role of oxygen in the effectiveness is still up for debate; however, multiple studies have shown that type I PDT performs better in comparison with type II PDT under low O_2_ concentration [25–28]. Additionally, Kolemen et al. have developed a remote-controlled release of ^1^O_2_, which overcomes the limitations of traditional PDT [29].

### 1.5. Effectiveness of PDT in Different Cancers

The approved application of PDT in the clinic so far is limited to precancerous keratosis skin lesions and some other nonmelanoma cancers [30]. Similarly, trials are ongoing to get the approval of PDT against esophageal, lung, and prostate cancers. Other indications against which PDT is being tried are breast, head and neck, bile duct, bladder, pancreas, cervix, brain, and some other cancers [10]. The first approval of PDT against cancer was using the photosensitizer “hematoporphyrin derivative” (HpD) against several cancers [31]. The active ingredient of HpD is porfimer sodium, which is often used in non-cutaneous solid tumors [31]. So far, it has been approved against bladder, esophageal, and non-small cell lung cancer (NSCLC) [32]. Porfimer sodium is mostly a harmless compound to tissues and is soluble in water. However, it causes sensitization of the skin against light [33] and can only be activated by specific wavelengths of light and needs further improvement [10]. Generally, PDT, although approved against solid cancer, is not widely used in practice and the revenue generated from PDT is fairly low in a clinic. In the next few paragraphs, the use of PDT against esophageal, lung, head and neck, skin, and some other cancer indications is described.

Cutaneous precancerous lesions or nonmelanoma skin cancers are the biggest indications for PDT. Actinic keratosis, Bowen disease, and basal cell carcinoma are treated with topical PDT in a noninvasive manner. PDT treatment has as good outcome as surgery in these cases in terms of recurrence of cancer. In addition, PDT does not cause scarring and has superior cosmetic outcomes [34]. Topical photosensitizer 5-aminolevulinic acid (ALA) and its derivatives are applied. Through the body's heme synthesis pathway, these compounds are converted to protoporphyrin IX (PPIX), which localizes to cancer sites because cancer tissues are better at taking up PPIX than normal tissues [35]. Typically, ALA is topically applied in regions of cancer, and blue light of 417 nm is shown for 17 minutes after 14–18 hours of photosensitizer application. Through various clinical trials, surrogate sensitizers and light range have been approved [10].

Another cancer often targeted by PDT is esophageal cancer [36]. PDT and local treatments are favorable in esophageal cancer because surgery often leads to postoperative complications [37]. In a number of clinical trials using porfimer sodium, high remission rates and 5-year survivals were observed for cancer of the esophagus [38]. Esophageal strictures and photosensitivity reactions were common adverse events associated with the treatments. Although PDT has been somewhat effective, other methods of treatment such as endoscopic submucosal dissection, radiofrequency ablation, and cryotherapy have been more effective at treating esophageal cancer. Hence, treatments using PDT have somewhat lost favor in the last few years [39].

Certain non-small cell lung cancers (NSCLCs) that are immune to all other treatments are treated with PDT [10]. Porfimer sodium PDT has been approved for microinvasive endobronchial NSCLC and entirely or partially obstructive endobronchial NSCLC [10]. External beam radiotherapy has been combined with PDT to clear obstruction in certain cases of NSCLC [40]. Additionally, bronchoscopy is used to target light to the exact tumor locations [10]. Several studies done using several different types of photosensitizer have shown remission of cancer; however, often there is re-emergence of cancer [10].

Similarly, head and neck squamous cell carcinoma (HNSCC) is another indication where PDT is widely tested [41]. Since this technique is often able to preserve surrounding nonneoplastic tissues, it has been favored for use in HNSCC. PDT is used to treat HNSCC early for curative reasons or in a later stage for palliative reasons [10]. Since HNSCC is very differently localized in different situations, a variety of illumination strategies, including surface and interstitial illumination with guided imaging, are possible [42]. Although different sensitizers have been tried at different stages of cancer, it is unlikely that PDT will replace surgery as a curative technique in the absence of further randomized control trials. ALA, porfimer sodium, and several other sensitizers have been tested in other cancer indications, including gliomas, bladder cancer, and prostate cancer with some promising results [10]. With the advent of more precise agents, research in and outside the clinic will continue for all these cancers.

### 1.6. Possible Areas of Improvement in Photodynamic Therapy

As we have discussed earlier, PDT has found application in a diverse range of solid tumors. Photosensitizers have been gradually improved over the years. PDT will get more effective when the production of singlet oxygen in space and time can be improved, which is possible at four key nodes: improvement in sensitizer excitation, improvement in sensitizer localization, improvement in sensitizer quenching, and improvement in scavenging singlet oxygen [6] (Figure 3).

Spatial and temporal control of PDT is possible through manipulation of sensitizer excitation. Diffraction of light places a limit on the resolution of light illumination. There are many techniques that focus the light higher than that allowed by the diffraction limit. One such method used to generate singlet oxygen is evanescent wave irradiation, achieved in a total internal reflection experiment. To achieve localized light focusing, the use of two-photon light is relevant. Often two photons are in a longer wavelength range, so the surrounding medium does not absorb the light. Only photosensitizers that can sequentially absorb two photons of light will be excited [6].

Most PDT agents absorb light in the visible range (400 to 700 nm wavelength) or near-infrared range (700 to 1350 nm wavelength). Diode lasers (630 to 1100 nm), dye lasers (390 to 1000 nm), alexandrite lasers (720 to 800 nm), and neodymium-doped yttrium aluminium garnet (Nd: YAG) lasers (1064 nm) are all available to excite sensitizers [43]. Optical parametric amplification or oscillation can generate NIR light source. A green tunable laser can be used to generate light of a longer wavelength. Target tissue can be irradiated using frontal diffuser fiber for radiation at the surface, multiple cylindrical diffuser fibers at the interstitial space, and balloon catheters for esophageal space [44–46].

There has been a lot of research to improve sensitizer quality and location. Photosensitizers can be classified into three generations based on their timeline of discovery [47]. The first generation of photosensitizer includes porfimer sodium (also called Photofrin) and hematoporphyrin. Naturally occurring porphyrins and their derivatives make up the first generation. There are several disadvantages of the first-generation photosensitizers, such as dark toxicity, low absorption in the red light, cutaneous phototoxicity, and issues related to hydrophobicity. These photosensitizers were further improved to give second-generation photosensitizers such as 5-aminolevulinic acid, chlorin, phthalocyanine, and benzoporphyrin derivative (verteporfin). These photosensitizers demonstrate lower phototoxicity, are cleared from the normal tissues faster, are activated by a shorter wavelength of light above 650 nm, and have a higher single oxygen quantum yield and solubility in water [47].

**Figure 4:**
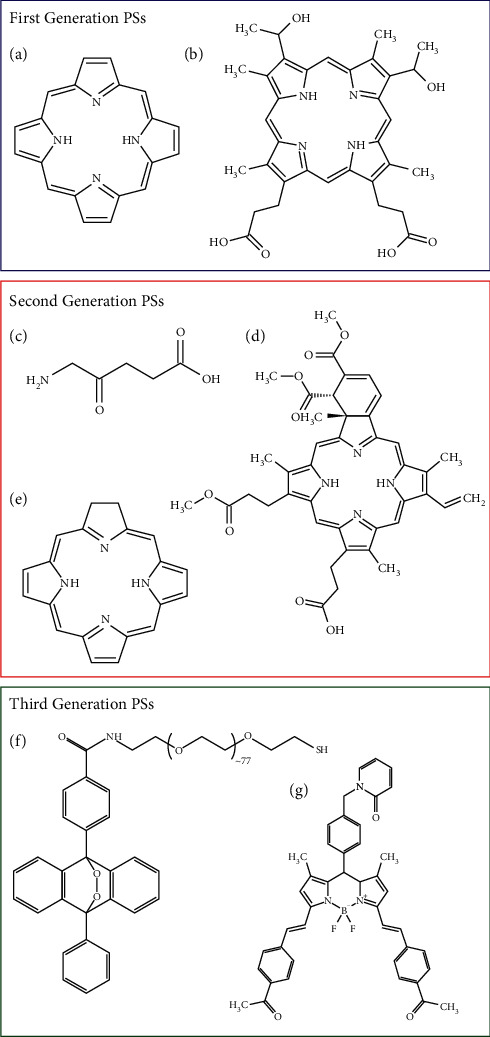
Examples of first-, second-, and third-generation photosensitizers with structure. (a) Porphyrin, (b) hematoporphyrin, (c) 5′-aminolevulinic acid, (d) chlorin, (e) verteporfin, (f) anthracene-9,10-endoperoxide derivative, and (g) 2-pydidone conjugated BODIPY.

To achieve better targeting and lower toxicity, third generation of photosensitizers is being developed. Available drugs are modified to create such compounds. Third-generation sensitizers are antibody conjugated and encapsulated into carriers to target specific areas. Potential insoluble photosensitizers are being transported in carbon and magnetic gold-based nanoparticles, liposomes, micelle, quantum dot, dendrimer, and polymer [47]. The hope is that next-generation photosensitizers are better than older-generation photosensitizers (Figure 4).

Photosensitizer targeting strategies can be passive and active [10]. The size and surface chemistry of nanoparticles carrying photosensitizers can be tuned so that they selectively accumulate in the tumor through enhanced permeability and retention (EPR) effects [48]. Tumors generally grow faster and consume local nutrients at a higher rate so that they end up taking more of our target molecules. They are also supposed to contain leaky pores in blood vessels that enhance the uptake of photosensitizer particles. Additionally, due to the imperfect lymphatic drainage system in tumors, less of the photosensitizers are lost [10]. Passive targeting involves the selection of nanoparticles based on shape, electric charge, hydrophilicity, and circulation time in the blood [49]. However, the relevance of EPR in actual human tumors versus the fast-growing tumor models in mice is questionable. Active targeting uses high-affinity ligands that specifically target cancer cells or tumor epithelial cells. Several ligands have been explored for their roles in targeting photosensitizers. These include peptides such as epidermal growth factor and arginine-glycine-aspartate peptide, proteins such as transferrin and antibodies, aptamers, vitamins, and carbohydrates [10, 50–52]. The role of monoclonal antibodies in targeting has also been explored in the clinic [53].

**Figure 5:**
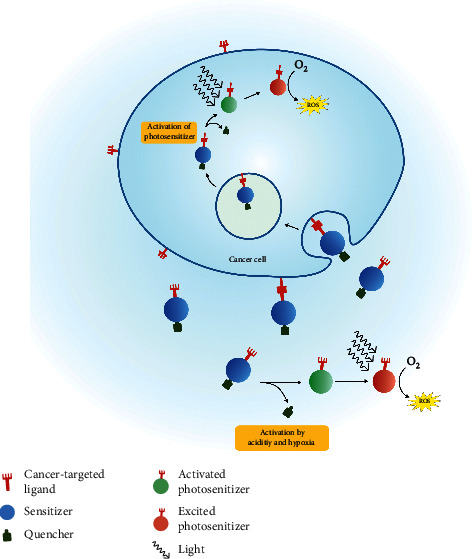
Process of ROS generation in a cancer cell due to photodynamic treatment. Photosensitizer (PS) specially designed for a particular cancer cell receptor will bind with it through the cancer target ligand. After recognizing the photosensitizer, the cancer cell takes it up through receptor-mediated endocytosis. The cellular environment inside the cancer cell is different from the extracellular space. The protease enzyme in the cell activates the engulfed PS by cleaving the quencher, and when the light is focused on the activated PS, it gets excited and converts the oxygen to ROS. ROS, such as singlet oxygen, causes cell death through several mechanisms. Sometimes, the sensitizer may be activated in the extracellular space due to acidity and hypoxia.

Quenching agent (Q) can be added along with sensitizer (S) to increase the specificity of singlet oxygen action in cancer cells. Normally, the electrons in the sensitizer relax, leading to the excitation of oxygen electrons to give a triplet oxygen state. Q can be fused to S in a changeable switch manner. In the blood or other tissues, excited electrons from S transfer onto Q due to absorption of light, and there is no creation of singlet oxygen species. Nevertheless, when Q is detached from S in cancer cells due to an acidic and/or hypoxic tumor microenvironment or action of proteases expressed by tumor cells, the promotion of electrons in S can lead to the formation of singlet oxygen, which can selectively kill cancer cells [6, 10] (Figure 5).

Similarly, there are selective quenchers of singlet oxygen that can be localized to regions other than tumors to decrease the side effects of singlet oxygen. In the same vein, after the activity of singlet oxygen is over, it can be quenched so that the activity does not spread over long distances. Among quenchers of singlet oxygen, water, sodium azide, and imidazole are the most prominent ones [6].

## 2. Mechanisms of Cell Death Caused by Singlet Oxygen

### 2.1. Catalase and Singlet Oxygen

Catalase is one of the most important antioxidant enzymes found in almost all aerobic organisms. It is a peroxisomal enzyme containing heme [54] and plays a key role in controlling the concentration of H_2_O_2_, which is produced particularly via the electron transport chain, and/or as a by-product of cellular metabolism, including protein folding [55–57]. Catalase is associated with the initiation of inflammation and aging, initiation of mutagenesis [58], apoptosis inhibition [59–62], and stimulation of a broad spectrum of tumors [63].

In comparison with normal cells, it has been reported that catalase expression in cancer tissues is altered. Some authors have observed an increase in catalase expression in tumors [64–66]. On the other hand, other studies have shown a decline in catalase expression in cancer cells [67–72]. In this context, a large body of evidence indicates that cancer cells are more sensitive to oxidative stress [73]. Most resistant cell lines (mesothelioma cell lines, HepG2 cells, WEHI 7.2 cells, etc.) showing high catalase expression are resistant to oxidative stress [74–76]. It has been found that inhibiting catalase activity using 3-aminotriazole (3-AT, specific inhibition of catalase) or catalase siRNA remarkably reduces the resistance of HepG2 and BT-20 cell lines to ROS [76, 77].

Chronic exposure to high H_2_O_2_ concentration or prooxidants has resulted in the generation of oxidative stress-resistant cells expressing high levels of catalase, as in Redox cell, leukemia, and fibroblast cell lines [78–80]. Exposure to high oxidative stress may have triggered the cell to increase catalase expression, which could be caused due to heritable changes in catalase gene dosage, transcription, translation, or a mutation in the coding region itself [81]. This phenomenon can also be observed in treatments related to anticancer drugs; increased levels of catalase were reported in oral cancer cells, bladder cancer cells, pancreatic cancer cells, and gastric cancer cells [82–85]. Furthermore, patients in a postoperative or/and postoperative chemotherapy stage showed a significant increase in catalase activity [86].

When exposed to singlet oxygen, catalase was susceptible to oxidative modification and damage, as indicated by the loss of activity [87–90]. Catalase plays a crucial role in removing the H_2_O_2_, so when the catalase within the cancer cells is deactivated or expressed in lower numbers, the cancer cells are significantly more sensitive to oxidative stress [91]. Thus, singlet oxygen can eliminate cancer cells by altering the activity of catalase enzyme.

### 2.2. Hypochlorous Acid (HOCl) Signaling

Hypochlorous acid (HOCl) functions as a potent antimicrobial agent and is a well-known physiological oxidant. It is enzymatically generated by the interaction of peroxidase (POD), H_2_O_2_, and chloride anions [92–94]. Besides having antimicrobial properties, it also has a vital role as a signaling molecule for oncogenesis control [95]. HOCl triggers immunogenic modulation (IM) through modification of antigens, which leads to immune response [96]. HOCl has been seen to increase HOCl-dependent tumor necrosis factor in peripheral blood mononuclear cells, which suggests that it contributes to activating signaling pathways in cells of the immune system leading to an inflammatory response [97].

A systematic review done by Han et al. indicates that an increase in NADPH oxidase (NOX) activity and expression is associated with tumorigenesis [98]. The production of superoxides correlates with the increase and decrease in NADH or NADPH, which is catalyzed by NADPH oxidase [99, 100].

H_2_O_2_ plays a vital role in tumor progression; however, it also seems essential in the antitumor mechanism [96]. It is formed by the dismutation of superoxide ions catalyzed by sodium oxide dismutase (SOD) [101]. The proliferation and maintenance of malignant phenotypes are driven by superoxide anions and H_2_O_2_ [95].

Myeloperoxidase (MPO) is a peroxidase enzyme abundantly expressed in neutrophils. It represents the “classical peroxidase (POD),” and it catalyzes the reaction between chloride and H_2_O_2_, generating HOCl [96, 102]. The chloride and H_2_O_2_ reaction catalyzed by MPO is a two-step reaction. Firstly, an H_2_O_2_ generated by the dismutation of superoxide, generated by NOX, reacts with a ferric MPO to form a compound I (MPO-I) and a water molecule. In a second step, MPO-I releases the oxygen in the presence of chloride and hydrogen ions to produce MPO and HOCl molecules.

(1) $$\begin{array}{c} \text{MPO}+{\text{H}}_{2}{\text{O}}_{2}⟶\text{MPO}-\text{I}+{\text{H}}_{2}\text{O,}\\ \text{MPO}-\text{I}+{\text{Cl}}^{-}+{\text{H}}^{+}⟶\text{MPO}+\text{HOCl}. \end{array}$$

HOCl triggers an immunogenic response and is linked to the attack of neutrophils on tumor cells [103–105]. However, at the later stage, cancer cells are resistant to HOCl-controlled death of cancer cells. The reason behind the control of HOCl signaling at later stages of tumor progression can be attributed to the membrane-associated catalase on the tumor cells [96]. Catalase intercepts the HOCl signaling pathway by eliminating the H_2_O_2_ [95]. H_2_O_2_ elimination constrains the interaction between the POD, H_2_O_2_, and halide system, thus reducing the HOCl production. Reduced HOCl is not able to induce immunogenic modulation. By reducing catalase expression level or inactivation of catalase, inhibition of HOCl signaling can be minimized.

**Figure 6:**
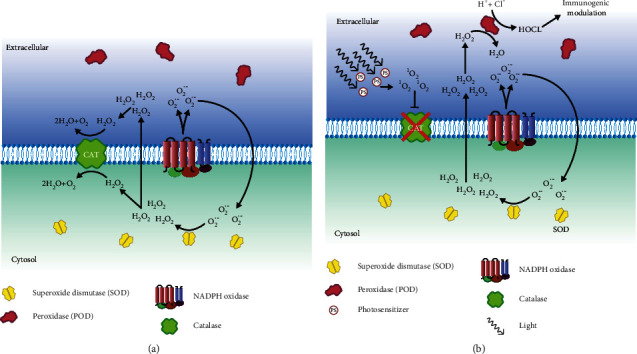
Activation and inhibition of the HOCl signaling pathway. HOCl signaling is induced in the plasma membrane of the cell. (a) Cancer cells may produce a higher amount of superoxides than normal cells. These superoxides are converted to hydrogen peroxide with the help of superoxide dismutase (SOD). Since hydrogen peroxide is toxic to the cell, the cancer cell produces membrane-bound catalase, which neutralizes the hydrogen peroxide. Hydrogen peroxide plays an essential role in HOCl signaling that causes cell death. (b) Singlet oxygen produced by the photodynamic therapy has been found to inhibit catalase activity, so in the absence of catalase activity, hydrogen peroxide piles up, which triggers peroxidase (POD) to initiate HOCl signaling. The HOCl signaling causes immunogenic modulation that triggers the immune system to eradicate cancer cells.

In photodynamic therapy, singlet oxygen is generated by light-induced excitation of photosensitizers [106]. Besides, singlet oxygen is also produced through biological reactions, such as the reaction between hydroperoxide and hypochlorite during phagocytosis [107]. Allen et al. in 1972 observed the production of singlet oxygen by phagocytes when stimulated with bacteria and exposed to radioactive decay of singlet oxygen [108]. The role of MPO/H_2_O_2_/Cl^−^ in the biological generation of singlet oxygen was confirmed by Khan in 2012 [109]. The relation between the singlet oxygen and HOCl is based on a positive feedback loop. By intervening with catalase, the singlet oxygen from an external source or a biological source helps auto-amplify singlet oxygen through HOCl [110] (Figure 6).

(2) H2O2+HOCl⟶O21+Cl−+H2O.

### 2.3. Apoptotic Pathways Induced by Singlet Oxygen

The mechanism of apoptosis is highly complex, involving a multitude of signaling molecules working in tandem to cause a molecular event. Our understanding of the process is still rudimentary, and research to date suggests that there are two major apoptotic pathways: the extrinsic or death receptor pathway and the intrinsic or mitochondrial pathway.

### 2.4. Extrinsic Pathway

Singlet oxygen is generated by two methods: photoactivation of photosensitizer and interaction between cell-derived H_2_O_2_ and peroxynitrite [111]. It stimulates the ligand-independent oligomerization of the Fas receptor, which binds with the adapter protein FADD [112]. Procaspase-8 binds with the FADD via the death effector domain, and this protein complex is called a death-inducing signaling complex (DISC), which autocatalyzes to activate procaspase-8 to caspase-8 [113]. Autoproteolysis of caspase-8 is essential for FAS-induced apoptosis. Mice with caspase-8 or a mutant of caspase-8 that cannot self-cleave were not able to go through Fas-induced apoptosis [114]. Active caspase-8 can also activate other caspase proteins, such as caspase-3 and caspase-7. Additionally, active caspase-8 causes the degradation of certain cellular proteins [115]. Caspase-3 catalyzes the cleavage of many vital cellular proteins and is the most frequently activated death protease [116]. It is activated during apoptosis [117, 118] and is responsible for the release of caspase-activated deoxyribonuclease (CAD) from inhibitor of caspase-activated DNase (ICAD) [119].

**Figure 7:**
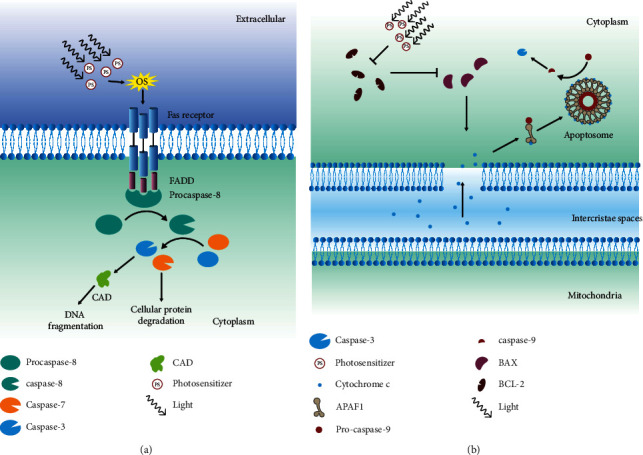
Extrinsic and intrinsic apoptotic pathways. (a) Oxidative stress induced due to photodynamic therapy (PDT) causes the activation of the Fas receptor, which binds with FADD followed by procaspase-8 to form a complex. This complex is called a death-inducing signaling complex (DISC), and it cleaves procaspase-8 into caspase-8. Caspase-8 cleaves caspase-3 and caspase-7. Caspase-3 releases caspase-activated deoxyribonuclease (CAD) from inhibitor of caspase-activated DNase (ICAD), which induces DNA fragmentation, whereas caspase-7 degrades the cellular proteins. (b) The activity of photosensitizer has been shown to contribute to the inhibition of Bcl-2 protein and increase in expression of BAX proteins. BAX increases mitochondrial outer membrane permeability, which causes the mitochondria to release cytochrome c into the cytoplasm. In the cytoplasm, cytochrome c binds with APAF1 and procaspase-9 to form apoptosomes. Apoptosome is responsible for the cleavage of caspase-9, which leads to the activation of caspase-3. Caspase-3 induces apoptosis via DNA fragmentation and cellular protein degradation.

Caspase-activated DNase (CAD) or DNA fragmentation factor subunit beta is a protein that breaks down the DNA during apoptosis. Activation of CAD induces inter-nucleosomal DNA degradation during apoptosis [119, 120] (Figure 7(a)).

### 2.5. Intrinsic Pathway

The intrinsic pathway involves a diverse range of non-receptor-mediated stimuli that evoke intercellular signaling, initiating mitochondrial events to induce apoptosis. The link between the singlet oxygen and mitochondrial associate apoptosis is not fully understood. Singlet oxygen can activate mitochondrial permeability transition (MPT) or inactivate it depending on the site [121]. In 1999, it was found that PDT targeted Bcl-2 protein [122, 123].

Bcl-2 family proteins control the permeability of the mitochondria membrane. Bax is a proapoptotic Bcl-2 family protein, and it increases the permeability of the mitochondria membrane, whereas Bcl-2 inhibits it [124]. Due to their opposing roles, the concentration of proteins determines the fate of the cell. When the cancer cell is exposed to the PDT, it triggers the destruction of Bcl-2 proteins, due to which it cannot interfere with Bax. Bax increases the permeability of the mitochondrial outer membrane, thus releasing ALF, endonuclease G, CAD, cytochrome c, and second mitochondria-derived activator of caspase (SMAC) into the cytoplasm [125]. These proteins activate the caspase-dependent and caspase-independent mitochondrial pathways. The released cytochrome C binds with apoptotic protease-activating factor 1 (APAF1), leading to the formation of apoptosome [126, 127]. It binds and activates procaspase-9 to caspase-9 [126, 128]. Caspase-9 activates the caspase-3 and caspase-7, leading to apoptosis [129] (Figure 7(b)).

SMAC works differently. It inhibits inhibitors of apoptosis proteins (IAPs) [130, 131]. Likewise, AIF gets translocated to the nucleus, where it causes DNA fragmentation and nuclear chromatin condensation [132].

### 2.6. Subcellular Singlet Oxygen and Cell Death

**Figure 8:**
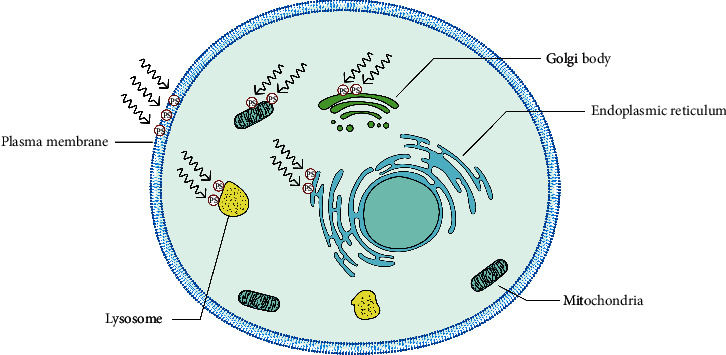
PDT can cause death by the localization of photosensitizer to different organelles. Photosensitizers located in various subcellular location induces cell death through different mechanisms. Normally, photosensitizers are targeted at the plasma membrane, Golgi body, lysosome, endoplasmic reticulum, and mitochondria to cause cell death. Generation of singlet oxygen via photodynamic therapy in the plasma membrane causes cell death through the HOCl signaling pathway (Figure 6) or through disruption of the plasma membrane, which causes cell swelling and rupture (Figure 9(a)). In the Golgi body, singlet oxygen plays a role upstream of mitochondria to cause mitochondria-dependent cell death. Disruption of the lysosome by singlet oxygen causes increased mitochondrial outer membrane permeability (Figure 10(a)), while photodynamic activities in the ER cause misfolding of protein, which triggers unfold protein response (URP), leading to activation of caspase-3 (Figure 10(b)).

At the cellular level, singlet oxygen can induce cell death via multiple subroutines that can be accidental or not. Localization of singlet oxygen generation will help determine the means of induced cell death. So, precise understanding of the effect of subcellular response due to singlet oxygen can be crucial for designing methods to efficiently eradicate tumor cells using photodynamic therapy. Precise localization of photosensitizers has been reported in select subcellular locations such as endoplasmic reticulum (ER), mitochondria, Golgi complex, lysosomes, and the plasma membrane [133, 134] (Figure 8).

### 2.7. Lysosome

The lysosome is an important cell organelle needed to process degrading and recycling cellular waste, for cellular signaling and for energy metabolism. Besides its role in cellular homeostasis, it also plays a crucial role in inducing lysosomal-dependent cell death (LDCD). Subcellular generation of singlet oxygen in lysosomes has been shown to cause the rapid release of lysosomal enzymes that activates caspases, leading to mitochondrial-mediated apoptosis [135–137]. Through the expulsion of degradative enzymes, singlet oxygen causes digestion of vital proteins and activation of caspase cascade [138]. Cathepsins B and D are the main proteases released after lysosomal membrane permeabilization (LMP), which causes the proteolytic activation of Bid, leading to caspase-dependent apoptosis [139, 140] (Figure 10(a)).

A Bcl-2 protein family is a group of proteins that shares BH (Bcl-2 homology) domains. They have been associated with apoptosis regulation, and these proteins can be divided into antiapoptotic and proapoptotic proteins. Some proteins only have the BH3 domain such as Bim, Bid, and Puma [124, 141]. Proapoptotic proteins, such as Bax and Bak, after activation, induce mitochondrial outer membrane permeability (MOMP), while antiapoptotic proteins such as Bcl-2 and Bcl-xl block the process. The activated Bid proteins interact with Bcl-2 to neutralize its effect so that the process of apoptosis continues [138]. MOMP is one of the important steps towards mitochondria-mediated apoptosis, and it is explained in the section on the intrinsic pathway.

### 2.8. Endoplasmic Reticulum (ER)

The endoplasmic reticulum (ER) is an essential cell organelle that synthesizes, folds, modifies, and transports proteins. Additionally, it also induces ER-related cell death when faced with irreversible ER stress. The full understanding of the involvement of singlet oxygen is still unknown; however, hypericin-based PDT has been shown to engender ROS-based ER stress or photooxidative (phox)-ER stress [142]. ER stress causes disturbance in ER proteostasis, which stimulates the unfolded protein response (UPR).

UPR is an important cellular response to ER stress. During ER stress, the folding capacity of ER is compromised, causing accumulation of unfolded protein, which is mitigated by UPR by refolding them. However, UPR can also be unsuccessful. When incorrect folding exceeds the threshold, cells commit to cell death [143]. Unfolding proteins in the ER initiate a stress signaling pathway via a stress sensor such as IRE1a. IRE1a recruits TRAF2 followed by procaspase-12, which forms IRE1a/TRAF2/caspase-12 complexes. This complex is able to activate caspase-12 [144]. Overexpression of IRE1a has been found to induce apoptosis related to caspase-12 [145], which activates caspases 9 and 3, which finally leads to caspase-dependent apoptosis. Besides, the recruitment of TRAF2 by IRE1a followed by ASK-1 activates the JNK signaling pathway, which also increases the caspase-12 activation [146] (Figure 10(b)).

Another central molecule associated with ER stress-induced apoptosis is C/EBP homologous protein (CHOP) and was the first molecule to be observed during ER stress [147]. ER stress induces increases in the transcription of CHOP. It was found that the overexpression of CHOP induces apoptosis [148], while CHOP-deficient cells were resistant to ER stress-induced apoptosis [149]. CHOP regulates the expression of Bcl-2 proteins when it binds with cAMP-responsive element-binding protein (CREB) [150]. This regulation of Bcl-2 helps increase the susceptibility of mitochondria towards proapoptotic BH3-only proteins such as Bax/Bak [151]. Sensitivity towards Bax/Bak also affects ER itself. Studies indicate that it alters calcium ion homeostasis [152, 153] and triggers calcium ion release during ER stress, which trickles down to activation of calpain and finally caspase-12 [151].

### 2.9. Plasma Membrane

The plasma membrane plays a crucial role in regulating the flow of the material in and out of the cell and acts as a gatekeeper. Its involvement in the two necrotic forms of regulated cell death (RCD) [154] can help us explain the molecular mechanism behind cell death caused by the photodynamic activation of subcellular localized photosensitizer in the plasma membrane.

The membrane-localized activation of photosensitizer has been shown to cause membrane disruption and successive necrosis-like cell death [155–158]. Morphological features associated with necrotic cell death are swelling of cell membranes, chromatin condensation, and subsequent rupture of a nucleus, organelles, and plasma membrane [159]. In a study done by Nakajima et al., minute plasma membrane perforations were observed, which were large enough for the entry and exit of ions but not for dextrans that were caused by photodamage. However, subsequently, longer photodamage to the plasma membrane after bleb formation leads to the entry of ethidium homodimer-1(EthD-1) (∼2.6 nm) and staining of the nucleus [157]. EthD-1 cannot penetrate the intact plasma membrane due to its size and charge. However, a damaged plasma membrane cannot hinder its entry, leading to staining of the nucleus.

**Figure 9:**
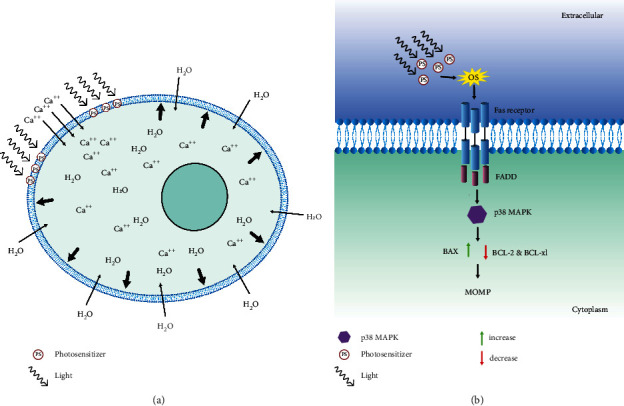
Cell swelling and p38 MAPK signaling. (a) Photosensitizer activity in the plasma membrane increases the permeability of the membrane, which causes the intake of calcium ions by the cell. The excess intake of the calcium ion causes the cytoplasmic content to be in the hypertonic state, which induces the absorption of water into the cell, thus leading to swelling of the cell and rupture of the cell. (b) p38 MAPK signaling initiates with activation of Fas receptor and formation of death-inducing signaling complex (DISC) complex. p38 MAPK induces the phosphorylation of Bcl-2 and BCL-xl, which are linked to the inhibition of apoptosis, and it has been found that p38 MAPK also increases the BAX expression. Deactivation of Bcl-2 and Bcl-xl and enhancement of the expression in BAX change the permeability of the mitochondrial outer membrane, which leads to activation of caspase-9 and apoptosis via the intrinsic pathway.

**Figure 10:**
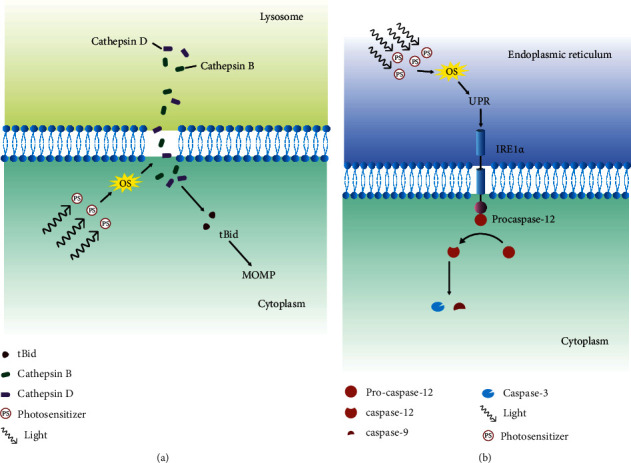
Cell death through lysosome membrane disruption and UPR pathway activation. (a) Damage caused by the oxidative stress due to photodynamic therapy in the lysosome membrane releases the cathepsins D and B into the cytoplasm. Cathepsins D and B cleave Bid into tBid, which increases the permeability of the mitochondria membrane. Change in the permeability induces mitochondria-dependent apoptosis. (b) Oxidative stress caused due to photodynamic therapy increases misfolding of the proteins in the endoplasmic reticulum. When the misfolding surpasses the threshold, it triggers unfolded protein response (UPR). UPR activates the IRE1a receptor that binds with procaspase-12 to cleave procaspase-12 to caspase-12. Caspase-12 induces the cleavage of caspase-9 and caspase-3, which leads to mitochondria-dependent apoptosis.

Even though cells are equipped with the plasma membrane repair mechanism, the long duration of photodamage causes irreversible disruption to the membrane and compromises the permeability of the cell membrane. Due to the lack of a barrier to entry, an increased inflow of calcium ions can activate cysteine proteases, which are responsible for the proteolysis of cytoskeletal protein. Besides, the ion inflow also disturbs the ionic imbalance leading to a surge of water inside the cell, causing swelling and, finally, cell rupture [160–163] (Figure 9(a)).

### 2.10. P38 MAPK Mediates Cell Death and Singlet Oxygen

Mitogen-activated protein kinases (MAPKs) are signaling components needed to communicate stimuli for a wide range of cellular responses. There are three subfamilies of MAPKs: extracellular signal-regulated kinases (ERKs), c-Jun N-terminal kinases (JNKs), and p38 mitogen-activated protein kinases. Among these, JNKs and p38 MAPKs are called stress-activated protein kinases, which are associated with stress-activated protein kinase pathways and activated by various stimuli such as UV irradiation, osmotic shock, and oxidants [164–166].

JNK and p38 kinase are both responsive to reactive oxygen species, but selective activation of p38 kinase has been reported in some cell systems [165, 167, 168]. For example, when HL-60 cells were treated with singlet oxygen, H_2_O_2_ causes rapid phosphorylation of p38; however, JNKs were not phosphorylated. P38 is necessary for apoptosis induced by singlet oxygen, whereas JNK is not. Similarly, p38 inhibition caused a partial reduction in the formation of DNA fragments induced by singlet oxygen [169].

Caspase-3 is a key protease behind the apoptosis induced by the singlet oxygen [170, 171] and the blockade of caspase-3 completely stopped the apoptosis, while it did not interfere with the p38 phosphorylation [169]. From this, we can conclude that caspase-3 acts downstream of the p38 kinase pathway.

Caspase-8 is one of the upstream components needed for the activation of the caspase cascade leading to the activation of caspase-3 during the singlet oxygen-induced apoptosis [172]. The linkage between the p38 and the caspase-8 was not found as inhibition of the p38 did not hinder the cleavage of caspase-8, while it reduced the caspase-3 cleavage and DNA fragmentation [169]. The involvement of p38 MAPK in receptor-induced cell death was poorly understood until the study conducted by Farley et al. They found that the p38 activated caspase-3 through mitochondrial-associated apoptosis rather than by direct activation [173].

Singlet oxygen has been found to stimulate the ligand-independent oligomerization of the Fas receptor, which binds with the adapter protein FADD [112]. The activation of p38 MAPK by the Fas receptor has been described in several studies [174]. In the study done by Farley et al., p38 MAPK contributed to Fas-induced cell death through phosphorylation of Bcl-2 and Bcl-xl and activation of caspase-9 [173]. In prior studies, it has been found that phosphorylation of Bcl-2 and Bcl-xl is associated with their inactivation [175–177]. In comparison with wild type, mutant MKK6 (Glu) CD8+ *t* cells showed strong phosphorylation of both Bcl-2 and Bcl-xl in the presence of p38 MAPK, thus preventing the mitochondrial accumulation of Bcl-2 and Bcl-xl [173]. Besides, during the PDT treatment of LoVo cells, the upregulation of Bax was also observed [178]. The downregulation of Bcl-2 and Bcl-xl and upregulation of Bax have a direct impact on the permeability of the mitochondrial membrane and the release of the cytochrome c (Figure 9(b)). The pathway followed by the cytochrome c to activate the caspases-3 is explained in the intrinsic pathway.

The activation of caspase-9 is linked to p38 MAPKs [173, 178]. The direct activation of caspase-9 by p38 MAPKs is not understood until now. Activation of caspase-9 is triggered by the release of cytochrome c due to the increase in permeability of the mitochondrial. The formation of Apaf/cytochrome c/procaspase-9 apoptotic complex causes cleavage of caspase-9 [179]. Caspase-9 is associated with the activation of caspase-3 and caspase-7, which finally leads to apoptosis [129].

### 2.11. Caspase 8 and Bid

Caspase-8 is a significant component in mitochondrial apoptosis [180, 181]. Activation of caspase-8 starts with the ligand-independent oligomerization of the Fas receptor, which binds with the adapter protein FADD [112], which is activated by the ROS such as singlet oxygen [111]. Procaspase-8 binds with the FADD to form DISC, which autocatalyzes to activate procaspase-8 to caspase-8 [113]. This activation of caspase-8 could activate caspase-3 directly or through the mitochondrial pathway by cleaving Bid [182].

Cleavage of Bid results in the formation of truncated Bid (tBid), which is capable of rapidly accumulating at mitochondria and causing MOMP [183, 184]. MOMP causes a release of cytochrome c. The release of cytochrome c causes mitochondria-mediated apoptosis, which is explained in the intrinsic pathway.

## 3. Concluding Remarks

PDT can be developed into an important arsenal against cancer; however, poor understanding of the underlying mechanism has hindered its optimal application. PDT has come a long way since its initial development. The generation of singlet oxygen and other reactive species can be improved through the advancement of chemical engineering of the new generation of sensitizers. Besides, advancement in understanding the biological signaling and process during the PDT is also quintessential for utilizing novel targets for better response.

Subcellular localization of the sensitizer has shown different responses depending on the location. These responses can be tied to the different signaling pathways triggered by the elicitor. Complete understandings of these signaling pathways are still lacking; however, deciphering these uncharted biochemical reactions can help us find novel targets for intercepting apoptosis and ways to neutralize cancer.

## References

[1] Smith S. L. (2017). War! what is it good for? mustard gas medicine. *Canadian Medical Association Journal*.

[2] Karran P., Attard N. (2008). Thiopurines in current medical practice: molecular mechanisms and contributions to therapy-related cancer. *Nature Reviews Cancer*.

[3] Giverhaug T., Loennechen T., Aarbakke J. (1999). The interaction of 6-mercaptopurine (6-MP) and methotrexate (MTX). *General Pharmacology: The Vascular System*.

[4] DeVita V. T., Chu E. (2008). A history of cancer chemotherapy. *Cancer Research*.

[5] Schiff L. J., Eisenberg W. C., Dziuba J., Taylor K., Moore S. J. (1987). Cytotoxic effects of singlet oxygen. *Environmental Health Perspectives*.

[6] Ogilby P. R. (2010). Singlet oxygen: there is indeed something new under the sun. *Chemical Society Reviews*.

[7] Uppal S., Banerjee B. (2021). Chemical bonding and structure. *Chemistry Part I (Reprinted)*.

[8] Atkins P., Paula J. D. (1961). Physical chemistry. *Journal of the Franklin Institute*.

[9] Laing M. (1989). The three forms of molecular oxygen. *Journal of Chemical Education*.

[10] Li X., Lovell J. F., Yoon J., Chen X. (2020). Clinical development and potential of photothermal and photodynamic therapies for cancer. *Nature Reviews Clinical Oncology*.

[11] Oleinick N. L., Evans H. H. (1998). The photobiology of photodynamic therapy: cellular targets and mechanisms. *Radiation Research*.

[12] Simpson C. R., Kohl M., Essenpreis M., Cope M. (1998). Near-infrared optical properties of ex vivo human skin and subcutaneous tissues measured using the monte carlo inversion technique. *Physics in Medicine and Biology*.

[13] Bolze F., Jenni S., Sour A., Heitz V. (2017). Molecular photosensitisers for two-photon photodynamic therapy. *Chemical Communications*.

[14] Fan W., Huang P., Chen X. (2016). Overcoming the achilles’ heel of photodynamic therapy. *Chemical Society Reviews*.

[15] Zebger I., Snyder J. W., Andersen L. K. (2007). Rapid communication: direct optical detection of singlet oxygen from a single cell. *Photochemistry and Photobiology*.

[16] Ogilby P. R., Foote C. S. (2002). Chemistry of singlet oxygen. 42. effect of solvent, solvent isotopic substitution, and temperature on the lifetime of singlet molecular oxygen (1.Delta.g). *Journal of the American Chemical Society*.

[17] Macpherson A. N., Truscott T. G., Turner P. H. (1994). Fourier-transform luminescence spectroscopy of solvated singlet oxygen. *Journal of the Chemical Society, Faraday Transactions*.

[18] Ackroyd R., Kelty C., Brown N., Reed M. (2007). The history of photodetection and photodynamic therapy. *Photochemistry and Photobiology*.

[19] Goldman L. (1966). A review: applications of the laser beam in cancer biology. *International Journal of Cancer*.

[20] Perry R. R., Smith P. D., Evans S., Pass H. I. (1991). Intravenous vs intraperitoneal sensitizer: implications for intraperitoneal photodynamic therapy. *Photochemistry and Photobiology*.

[21] Wang Y.-Y., Liu Y.-C., Sun H., Guo D.-S. (2019). Type I photodynamic therapy by organic–inorganic hybrid materials: from strategies to applications. *Coordination Chemistry Reviews*.

[22] Chen D., Xu Q., Wang W., Shao J., Huang W., Dong X. (2021). Type I photosensitizers revitalizing photodynamic oncotherapy. *Small*.

[23] Dos Santos A. F., De Almeida D. R. Q., Terra L. F., Baptista M. S., Labriola L. (2019). Photodynamic therapy in cancer treatment—an update review. *Journal of Cancer Metastasis and Treatment*.

[24] Li X., Kwon N., Guo T., Liu Z., Yoon J. (2018). Innovative strategies for hypoxic tumor photodynamic therapy. *Angewandte Chemie International Edition*.

[25] Ding H., Yu H., Dong Y. (2011). Photoactivation switch from type II to type I reactions by electron-rich micelles for improved photodynamic therapy of cancer cells under hypoxia. *Journal of Controlled Release*.

[26] Gilson R. C., Black K. C. L., Lane D. D., Achilefu S. (2017). Hybrid TiO_2_-ruthenium nano-photosensitizer synergistically produces reactive oxygen species in both hypoxic and normoxic conditions. *Angewandte Chemie International Edition*.

[27] Lv Z., Wei H., Li Q. (2018). Achieving efficient photodynamic therapy under both normoxia and hypoxia using cyclometalated Ru (ii) photosensitizer through type I photochemical process. *Chemical Science*.

[28] Wang X., Hu J., Wang P. (2015). Analysis of the in vivo and in vitro effects of photodynamic therapy on breast cancer by using a sensitizer, sodium. *Theranostics*.

[29] Kolemen S., Ozdemir T., Lee D. (2016). Remote-controlled release of singlet oxygen by the plasmonic heating of endoperoxide-modified gold nanorods: towards a paradigm change in photodynamic therapy. *Angewandte Chemie International Edition*.

[30] Wan M. T., Lin J. Y. (2014). Current evidence and applications of photodynamic therapy in dermatology. *Clinical, Cosmetic and Investigational Dermatology*.

[31] Dougherty T. J., Kaufman J. E., Goldfarb A., Weishaupt K. R., Boyle D., Mittleman A. (1978). Photoradiation therapy for the treatment of malignant tumors. *Cancer Research*.

[32] Allison R. R., Mota H. C., Sibata C. H. (2004). Clinical PD/PDT in North America: an historical review. *Photodiagnosis and Photodynamic Therapy*.

[33] Dougherty T. J., Cooper M. T., Mang T. S. (1990). Cutaneous phototoxic occurrences in patients receiving photofrin. *Lasers in Surgery and Medicine*.

[34] Morton C. A., Szeimies R.-M., Basset-Seguin N. (2019). European dermatology forum guidelines on topical photodynamic therapy 2019 part 1: treatment delivery and established indications—actinic keratoses, bowen’s disease and basal cell carcinomas. *Journal of the European Academy of Dermatology and Venereology*.

[35] Ohgari Y., Nakayasu Y., Kitajima S. (2005). Mechanisms involved in *δ*-aminolevulinic acid (ALA)-induced photosensitivity of tumor cells: relation of ferrochelatase and uptake of ALA to the accumulation of protoporphyrin. *Biochemical Pharmacology*.

[36] Qumseya B. J., David W., Wolfsen H. C. (2013). Photodynamic therapy for barrett’s esophagus and esophageal carcinoma. *Clinical Endoscopy*.

[37] Ferguson M. K., Martin T. R., Reeder L. B., Olak J. (1997). Mortality after esophagectomy: risk factor analysis. *World Journal of Surgery*.

[38] Overholt B. F., Lightdale C. J., Wang K. K. (2005). Photodynamic therapy with porfimer sodium for ablation of high-grade dysplasia in Barrett’s esophagus: international, partially blinded, randomized phase III trial. *Gastrointestinal Endoscopy*.

[39] Wu H., Minamide T., Yano T. (2019). Role of photodynamic therapy in the treatment of esophageal cancer. *Digestive Endoscopy*.

[40] Lam S., Kostashuk E. C., Coy E. P. (1987). A randomized comparative study of the safety and efficacy of photodynamic therapy using photofrin II combined with palliative radiotherapy versus palliative radiotherapy alone in patients with inoperable obstructive non-small cell bronchogenic carcinoma. *Photochemistry and Photobiology*.

[41] Biel M. A. (2010). Photodynamic therapy of head and neck cancers. *Methods in Molecular Biology*.

[42] Jerjes W., Upile T., Akram S., Hopper C. (2010). The surgical palliation of advanced head and neck cancer using photodynamic therapy. *Clinical Oncology*.

[43] Lin J.-T. (2016). Progress of medical lasers: fundamentals and applications. *Medical Devices and Diagnostic Engineering*.

[44] Panjehpour M., Overholt B. F., Denovo R. C., Sneed R. E., Petersen M. G. (1992). Centering balloon to improve esophageal photodynamic therapy. *Lasers in Surgery and Medicine*.

[45] Shafirstein G., Bellnier D., Oakley E. (2017). Interstitial photodynamic therapy—a focused review. *Cancers*.

[46] van den Bergh H. (1998). On the evolution of some endoscopic light delivery systems for photodynamic therapy. *Endoscopy*.

[47] Gunaydin G., Gedik M. E., Ayan S. (2021). Photodynamic therapy for the treatment and diagnosis of cancer–a review of the current clinical status. *Frontiers of Chemistry*.

[48] Fang J., Nakamura H., Maeda H. (2011). The EPR effect: unique features of tumor blood vessels for drug delivery, factors involved, and limitations and augmentation of the effect. *Advanced Drug Delivery Reviews*.

[49] Bertrand N., Wu J., Xu X., Kamaly N., Farokhzad O. C. (2014). Cancer nanotechnology: the impact of passive and active targeting in the era of modern cancer biology. *Advanced Drug Delivery Reviews*.

[50] Conde J., Oliva N., Zhang Y., Artzi N. (2016). Local triple-combination therapy results in tumour regression and prevents recurrence in a colon cancer model. *Nature Materials*.

[51] Kotagiri N., Sudlow G. P., Akers W. J., Achilefu S. (2015). Breaking the depth dependency of phototherapy with cerenkov radiation and low-radiance-responsive nanophotosensitizers. *Nature Nanotechnology*.

[52] Li Z., Huang P., Zhang X. (2009). RGD-conjugated dendrimer-modified gold nanorods for in vivo tumor targeting and photothermal therapy^†^. *Molecular Pharmaceutics*.

[53] Mew D., Wat C. K., Towers G. H., Levy J. G. (1983). Photoimmunotherapy: treatment of animal tumors with tumor-specific monoclonal antibody-hematoporphyrin conjugates. *The Journal of Immunology*.

[54] Putnam C. D., Arvai A. S., Bourne Y., Tainer J. A. (2000). Active and inhibited human catalase structures: ligand and NADPH binding and catalytic mechanism. *Journal of Molecular Biology*.

[55] Gaetani G. F., Ferraris A. M., Rolfo M., Mangerini R., Arena S., Kirkman H. N. (1996). Predominant role of catalase in the disposal of hydrogen peroxide within human erythrocytes. *Blood*.

[56] Lennicke C., Rahn J., Lichtenfels R., Wessjohann L. A., Seliger B. (2015). Hydrogen peroxide—production, fate and role in redox signaling of tumor cells. *Cell Communication and Signaling*.

[57] Mueller S., Riedel H. D., Stremmel W. (1997). Direct evidence for catalase as the predominant H_2_O_2_-removing enzyme in human erythrocytes. *Blood*.

[58] Vuillaume M. (1987). Reduced oxygen species, mutation, induction and cancer initiation. *Mutation Research: Reviews in Genetic Toxicology*.

[59] Bai J., Cederbaum A. I. (2003). Catalase protects HepG2 cells from apoptosis induced by DNA-damaging agents by accelerating the degradation of p53. *Journal of Biological Chemistry*.

[60] Islam K. N., Kayanoki Y., Kaneto H. (1997). TGF-*β*1 triggers oxidative modifications and enhances apoptosis in hit cells through accumulation of reactive oxygen species by suppression of catalase and glutathione peroxidase. *Free Radical Biology and Medicine*.

[61] Sandstrom P. A., Buttke T. M. (1993). Autocrine production of extracellular catalase prevents apoptosis of the human CEM T-cell line in serum-free medium. *Proceedings of the National Academy of Sciences*.

[62] Yabuki M., Kariya S., Ishisaka R. (1999). Resistance to nitric oxide-mediated apoptosis in HL-60 variant cells is associated with increased activities of Cu, Zn-superoxide dismutase and catalase. *Free Radical Biology and Medicine*.

[63] Miyamoto T., Hayashi M., Takeuchi A. (1996). Identification of a novel growth-promoting factor with a wide target cell spectrum from various tumor cells as catalase. *Journal of Biochemistry*.

[64] Hwang T. S., Choi H. K., Han H. S. (2007). Differential expression of manganese superoxide dismutase, copper/zinc superoxide dismutase, and catalase in gastric adenocarcinoma and normal gastric mucosa. *European Journal of Surgical Oncology*.

[65] Rainis T., Maor I., Lanir A., Shnizer S., Lavy A. (2007). Enhanced oxidative stress and leucocyte activation in neoplastic tissues of the colon. *Digestive Diseases and Sciences*.

[66] Sander C. S., Hamm F., Elsner P., Thiele J. J. (2003). Oxidative stress in malignant melanoma and non-melanoma skin cancer. *British Journal of Dermatology*.

[67] Baker A. M., Oberley L. W., Cohen M. B. (1997). Expression of antioxidant enzymes in human prostatic adenocarcinoma. *The Prostate*.

[68] Cullen J. J., Mitros F. A., Oberley L. W. (2003). Expression of antioxidant enzymes in diseases of the human pancreas: another link between chronic pancreatitis and pancreatic cancer. *Pancreas*.

[69] Chung-Man Ho J., Zheng S., Comhair S. A. A., Farver C., Erzurum S. C. (2001). Differential expression of manganese superoxide dismutase and catalase in lung cancer. *Cancer Research*.

[70] Kwei K. A., Finch J. S., Thompson E. J., Bowden G. T. (2004). Transcriptional repression of catalase in mouse skin tumor progression. *Neoplasia*.

[71] Lauer C., Völkl A., Riedl S., Fahimi H. D., Beier K. (1999). Impairment of peroxisomal biogenesis in human colon carcinoma. *Carcinogenesis*.

[72] Marklund S. L., Westman N. G., Lundgren E., Roos G. (1982). Copper- and zinc-containing superoxide dismutase, manganese-containing superoxide dismutase, catalase, and glutathione peroxidase in normal and neoplastic human cell lines and normal human tissues. *Cancer Research*.

[73] Glorieux C., Zamocky M., Sandoval J. M., Verrax J., Calderon P. B. (2015). Regulation of catalase expression in healthy and cancerous cells. *Free Radical Biology and Medicine*.

[74] Kahlos K., Soini Y., Sormunen R. (2001). Expression and prognostic significance of catalase in malignant mesothelioma. *Cancer*.

[75] Tome M. E., Baker A. F., Powis G., Payne C. M., Briehl M. M. (2001). Catalase-overexpressing thymocytes are resistant to glucocorticoid-induced apoptosis and exhibit increased net tumor growth. *Cancer Research*.

[76] Zhao M. X., Wen J. L., Wang L., Wang X. P., Chen T. S. (2019). Intracellular catalase activity instead of glutathione level dominates the resistance of cells to reactive oxygen species. *Cell Stress & Chaperones*.

[77] Klingelhoeffer C., Kämmerer U., Koospal M. (2012). Natural resistance to ascorbic acid induced oxidative stress is mainly mediated by catalase activity in human cancer cells and catalase-silencing sensitizes to oxidative stress. *BMC Complementary and Alternative Medicine*.

[78] Glorieux C., Calderon P. B. (2018). Catalase down-regulation in cancer cells exposed to arsenic trioxide is involved in their increased sensitivity to a pro-oxidant treatment. *Cancer Cell International*.

[79] Hunt C. R., Sim J. E., Sullivan S. J. (1998). Genomic instability and catalase gene amplification induced by chronic exposure to oxidative stress. *Cancer Research*.

[80] Yamada M., Hashinaka K., Inazawa J., Abe T. (1991). Expression of catalase and myeloperoxidase genes in hydrogen peroxide-resistant HL-60 cells. *DNA and Cell Biology*.

[81] Akman S. A., Forrest G., Chu F. F., Doroshow J. H. (1989). Resistance to hydrogen peroxide associated with altered catalase mRNA stability in MCF7 breast cancer cells. *Biochimica et Biophysica Acta (BBA)—Gene Structure and Expression*.

[82] Kuramitsu Y., Taba K., Ryozawa S. (2010). Identification of up- and down-regulated proteins in gemcitabine-resistant pancreatic cancer cells using two-dimensional gel electrophoresis and mass spectrometry. *Anticancer Research*.

[83] Xu B. H., Gupta V., Singh S. V. (1994). Characterization of a human bladder cancer cell line selected for resistance to mitomycin C. *International Journal of Cancer*.

[84] Xu H., Choi S. M., An C. S. (2005). Concentration-dependent collateral sensitivity of cisplatin-resistant gastric cancer cell sublines. *Biochemical and Biophysical Research Communications*.

[85] Yen H. C., Li S. H., Majima H. J. (2011). Up-regulation of antioxidant enzymes and coenzyme Q10 in a human oral cancer cell line with acquired bleomycin resistance. *Free Radical Research*.

[86] Rajneesh C. P., Manimaran A., Sasikala K. R., Adaikappan P. (2008). Lipid peroxidation and antioxidant status in patients with breast cancer. *Singapore Medical Journal*.

[87] Bauer G., Sersenová D., Graves D. B., Machala Z. (2019). Dynamics of singlet oxygen-triggered, RONS-based apoptosis induction after treatment of tumor cells with cold atmospheric plasma or plasma-activated medium. *Scientific Reports*.

[88] Escobar J. A., Rubio M. A., Lissi E. A. (1996). SOD and catalase inactivation by singlet oxygen and peroxyl radicals. *Free Radical Biology and Medicine*.

[89] Kim S. Y., Kwon O. J., Park J. W. (2001). Inactivation of catalase and superoxide dismutase by singlet oxygen derived from photoactivated dye. *Biochimie*.

[90] Riethmüller M., Burger N., Bauer G. (2015). Singlet oxygen treatment of tumor cells triggers extracellular singlet oxygen generation, catalase inactivation and reactivation of intercellular apoptosis-inducing signaling. *Redox Biology*.

[91] Glorieux C., Sandoval J. M., Dejeans N. (2018). Evaluation of potential mechanisms controlling the catalase expression in breast cancer cells. *Oxidative Medicine and Cellular Longevity*.

[92] Kettle A. J., Van Dalen C. J., Winterbourn C. C. (1997). Peroxynitrite and myeloperoxidase leave the same footprint in protein nitration. *Redox Report*.

[93] Kettle A. J., Winterbourn C. C. (1997). Myeloperoxidase: a key regulator of neutrophil oxidant production. *Redox Report*.

[94] Klebanoff S. J. (2005). Myeloperoxidase: friend and foe. *Journal of Leukocyte Biology*.

[95] Bauer G. (2018). HOCl and the control of oncogenesis. *Journal of Inorganic Biochemistry*.

[96] Bauer G. (2017). SiRNA-based analysis of the abrogation of the protective function of membrane-associated catalase of tumor cells. *Anticancer Research*.

[97] Schieven G. L., De Fex H., Stephenson L. (2002). Hypochlorous acid activates tyrosine phosphorylation signal pathways leading to calcium signaling and TNF*α*Production. *Antioxidants and Redox Signaling*.

[98] Han M., Zhang T., Yang L., Wang Z., Ruan J., Chang X. (2016). Association between NADPH oxidase (NOX) and lung cancer: a systematic review and meta-analysis. *Journal of Thoracic Disease*.

[99] Irani K., Goldschmidt-Clermont P. J. (1998). Ras, superoxide and signal transduction. *Biochemical Pharmacology*.

[100] Meier B., Radeke H. H., Selle S. (1989). Human fibroblasts release reactive oxygen species in response to interleukin-1 or tumour necrosis factor-*α*. *Biochemical Journal*.

[101] Oberley L. W., Buettner G. R. (1979). Role of superoxide dismutase in cancer: a review. *Cancer Research*.

[102] Mika D., Guruvayoorappan C. (2011). Myeloperoxidase: the yin and yang in tumour progression. *Journal of Experimental Therapeutics and Oncology*.

[103] Clark R. A., Klebanoff S. J. (1975). Neutrophil-mediated tumor cell cytotoxicity: role of the peroxidase system. *Journal of Experimental Medicine*.

[104] Clark R. A., Klebanoff S. J. (1979). Chemotactic factor inactivation by the myeloperoxidase-hydrogen peroxide-halide system. *Journal of Clinical Investigation*.

[105] Clark R. A., Szot S. (1981). The myeloperoxidase-hydrogen peroxide-halide system as effector of neutrophil-mediated tumor cell cytotoxicity. *The Journal of Immunology*.

[106] Foote C. S. (1968). Mechanisms of photosensitized oxidation. *Science*.

[107] Rosen H., Klebanoff S. J. (1977). Formation of singlet oxygen by the myeloperoxidase-mediated antimicrobial system. *Journal of Biological Chemistry*.

[108] Allen R. C., Stjernholm R. L., Steele R. H. (1972). Evidence for the generation of an electronic excitation state(s) in human polymorphonuclear leukocytes and its participation in bactericidal activity. *Biochemical and Biophysical Research Communications*.

[109] Khan A. Q., Khan R., Qamar W. (2012). Caffeic acid attenuates 12-O-tetradecanoyl-phorbol-13-acetate (TPA)-induced NF-*κ*B and COX-2 expression in mouse skin: abrogation of oxidative stress, inflammatory responses and proinflammatory cytokine production. *Food and Chemical Toxicology*.

[110] Bauer G. (2016). The antitumor effect of singlet oxygen. *Anticancer Research*.

[111] Di Mascio P., Bechara E. J. H., Medeiros M. H. G., Briviba K., Sies H. (1994). Singlet molecular oxygen production in the reaction of peroxynitrite with hydrogen peroxide. *FEBS Letters*.

[112] Zhuang S., Demirs J. T., Kochevar I. E. (2001). Protein kinase C inhibits singlet oxygen-induced apoptosis by decreasing caspase-8 activation. *Oncogene*.

[113] Kischkel F. C., Hellbardt S., Behrmann I. (1995). Cytotoxicity-dependent APO-1 (fas/CD95)-associated proteins form a death-inducing signaling complex (DISC) with the receptor. *The EMBO Journal*.

[114] Kang T.-B., Oh G.-S., Scandella E. (2008). Mutation of a self-processing site in caspase-8 compromises its apoptotic but not its functions in bacterial artificial chromosome-transgenic mice. *The Journal of Immunology*.

[115] Stennicke H. R., Jürgensmeier J. M., Shin H. (1998). Pro-caspase-3 is a major physiologic target of caspase-8. *Journal of Biological Chemistry*.

[116] Porter A. G., Jänicke R. U. (1999). Emerging roles of caspase-3 in apoptosis. *Cell Death & Differentiation*.

[117] Enari M., Talanian R. V., Wrong W. W., Nagata S. (1996). Sequential activation of ICE-like and CPP32-like proteases during fas-mediated apoptosis. *Nature*.

[118] Henkart P. A. (1996). Ice family proteases: mediators of all apoptotic cell death?. *Immunity*.

[119] Sakahira H., Enari M., Nagata S. (1998). Cleavage of CAD inhibitor in CAD activation and DNA degradation during apoptosis. *Nature*.

[120] Enari M., Sakahira H., Yokoyama H., Okawa K., Iwamatsu A., Nagata S. (1998). A caspase-activated DNase that degrades DNA during apoptosis, and its inhibitor ICAD. *Nature*.

[121] Moreno G., Poussin K., Ricchelli F., Salet C. (2001). The effects of singlet oxygen produced by photodynamic action on the mitochondrial permeability transition differ in accordance with the localization of the sensitizer. *Archives of Biochemistry and Biophysics*.

[122] Kim H. R., Luo Y., Li G., Kessel D. (1999). Enhanced apoptotic response to photodynamic therapy after-2 transfection. *Cancer Research*.

[123] Xue L. Y., Chiu S. M., Oleinick N. L. (2001). Photochemical destruction of the Bcl-2 oncoprotein during photodynamic therapy with the phthalocyanine photosensitizer Pc 4. *Oncogene*.

[124] Youle R. J., Strasser A. (2008). The BCL-2 protein family: opposing activities that mediate cell death. *Nature Reviews Molecular Cell Biology*.

[125] Muñoz-Pinedo C., Guío-Carrión A., Goldstein J. C., Fitzgerald P., Newmeyer D. D., Green D. R. (2006). Different mitochondrial intermembrane space proteins are released during apoptosis in a manner that is coordinately initiated but can vary in duration. *Proceedings of the National Academy of Sciences*.

[126] Li P., Zhou L., Zhao T. (2017). Caspase-9: structure, mechanisms and clinical application. *Oncotarget*.

[127] Tait S. W. G., Green D. R. (2010). Mitochondria and cell death: outer membrane permeabilization and beyond. *Nature Reviews Molecular Cell Biology*.

[128] Ow Y.-L. P., Green D. R., Hao Z., Mak T. W. (2008). Cytochrome c: functions beyond respiration. *Nature Reviews Molecular Cell Biology*.

[129] Lakhani S. A., Masud A., Kuida K. (2006). Caspases 3 and 7: key mediators of mitochondrial events of apoptosis. *Science*.

[130] Schimmer A. D. (2004). Inhibitor of apoptosis proteins: translating basic knowledge into clinical practice. *Cancer Research*.

[131] Van Loo G., Van Gurp M., Depuydt B. (2002). The serine protease Omi/HtrA2 is released from mitochondria during apoptosis. Omi interacts with caspase-inhibitor XIAP and induces enhanced caspase activity. *Cell Death & Differentiation*.

[132] Joza N., Susin S. A., Daugas E. (2001). Essential role of the mitochondrial apoptosis-inducing factor in programmed cell death. *Nature*.

[133] Abrahamse H., Hamblin M. R. (2016). New photosensitizers for photodynamic therapy. *Biochemical Journal*.

[134] Van Straten D., Mashayekhi V., De Bruijn H. S., Oliveira S., Robinson D. J. (2017). Oncologic photodynamic therapy: basic principles, current clinical status and future directions. *Cancers*.

[135] Caruso J. A., Mathieu P. A., Reiners J. J. (2005). Sphingomyelins suppress the targeted disruption of lysosomes/endosomes by the photosensitizer NPe6 during photodynamic therapy. *Biochemical Journal*.

[136] Kessel D., Luo Y., Mathieu P., Reiners J. J. (2000). Determinants of the apoptotic response to lysosomal photodamage. *Photochemistry and Photobiology*.

[137] Reiners J. J., Caruso J. A., Mathieu P., Chelladurai B., Yin X. M., Kessel D. (2002). Release of cytochrome c and activation of pro-caspase-9 following lysosomal photodamage involves bid cleavage. *Cell Death & Differentiation*.

[138] Boya P., Kroemer G. (2008). Lysosomal membrane permeabilization in cell death. *Oncogene*.

[139] Cirman T., Oresić K., Mazovec G. D. (2004). Selective disruption of lysosomes in HeLa cells triggers apoptosis mediated by cleavage of Bid by multiple papain-like lysosomal cathepsins. *Journal of Biological Chemistry*.

[140] Uchimoto T., Nohara H., Kamehara R., Iwamura M., Watanabe N., Kobayashi Y. (1999). Mechanism of apoptosis induced by a lysosomotropic agent, L-leucyl-L-leucine methyl ester. *Apoptosis: An International Journal on Programmed Cell Death*.

[141] Giam M., Huang D. C. S., Bouillet P. (2008). BH3-only proteins and their roles in programmed cell death. *Oncogene*.

[142] Garg A. D., Agostinis P. (2014). ER stress, autophagy and immunogenic cell death in photodynamic therapy-induced anti-cancer immune responses. *Photochemical and Photobiological Sciences*.

[143] Kim R., Emi M., Tanabe K., Murakami S. (2006). Role of the unfolded protein response in cell death. *Apoptosis*.

[144] Yoneda T., Imaizumi K., Oono K. (2001). Activation of caspase-12, an endoplastic reticulum (ER) resident caspase, through tumor necrosis factor receptor-associated factor 2-dependent mechanism in response to the ER stress. *Journal of Biological Chemistry*.

[145] Wang X., Ron D. (1996). Stress-induced phosphorylation and activation of the transcription factor CHOP (GADD153) by p38 MAP kinase. *Science*.

[146] Urano F., Wang X., Bertolotti A. (2000). Coupling of stress in the ER to activation of JNK protein kinases by transmembrane protein kinase IRE1. *Science*.

[147] Oyadomari S., Mori M. (2004). Roles of CHOP/GADD153 in endoplasmic reticulum stress. *Cell Death & Differentiation*.

[148] Kim D.-G., You K.-R., Liu M.-J., Choi Y.-K., Won Y.-S. (2002). GADD153-mediated anticancer effects of N-(4-hydroxyphenyl)retinamide on human hepatoma cells. *Journal of Biological Chemistry*.

[149] Zinszner H., Kuroda M., Wang X. (1998). CHOP is implicated in programmed cell death in response to impaired function of the endoplasmic reticulum. *Genes & Development*.

[150] McCullough K. D., Martindale J. L., Klotz L. O., Aw T. Y., Holbrook N. J. (2001). Gadd153 sensitizes cells to endoplasmic reticulum stress by down-regulating Bcl2 and perturbing the cellular redox state. *Molecular and Cellular Biology*.

[151] Kim R., Emi M., Tanabe K. (2006). Role of mitochondria as the gardens of cell death. *Cancer Chemotherapy and Pharmacology*.

[152] Scorrano L., Oakes S. A., Opferman J. T. (2003). BAX and bak regulation of endoplasmic reticulum Ca2+: a control point for apoptosis. *Science*.

[153] Zong W.-X., Li C., Hatzivassiliou G. (2003). Bax and Bak can localize to the endoplasmic reticulum to initiate apoptosis. *Journal of Cell Biology*.

[154] Kolbrink B., Riebeling T., Kunzendorf U., Krautwald S. (2020). Plasma membrane pores drive inflammatory cell death. *Frontiers in Cell and Developmental Biology*.

[155] Ahn W. S., Bae S. M., Huh S. W. (2004). Necrosis-like death with plasma membrane damage against cervical cancer cells by photodynamic therapy. *International Journal of Gynecological Cancer: Official Journal of the International Gynecological Cancer Society*.

[156] Kim J., Santos O. A., Park J.-H. (2014). Selective photosensitizer delivery into plasma membrane for effective photodynamic therapy. *Journal of Controlled Release*.

[157] Nakajima K., Takakura H., Shimizu Y., Ogawa M. (2018). Changes in plasma membrane damage inducing cell death after treatment with near-infrared photoimmunotherapy. *Cancer Science*.

[158] Thompson S. A., Aggarwal A., Singh S., Adam A. P., Tome J. P. C., Drain C. M. (2018). Compromising the plasma membrane as a secondary target in photodynamic therapy-induced necrosis. *Bioorganic & Medicinal Chemistry*.

[159] Wyllie A. H., Kerr J. F., Currie A. R. (1980). Cell death: the significance of apoptosis. *International Review of Cytology*.

[160] Fink S. L., Cookson B. T. (2005). Apoptosis, pyroptosis, and necrosis: mechanistic description of dead and dying eukaryotic cells. *Infection and Immunity*.

[161] Liu X., Van Vleet T., Schnellmann R. G. (2004). The role of calpain in oncotic cell death. *Annual Review of Pharmacology and Toxicology*.

[162] Okada Y., Sato K., Numata T. (2009). Pathophysiology and puzzles of the volume-sensitive outwardly rectifying anion channel. *The Journal of Physiology*.

[163] Trump B. F., Berezesky I. K. (1996). The role of altered [Ca^2+^]_i_ regulation in apoptosis, and necrosis. *Biochimica et Biophysica Acta (BBA)—Molecular Cell Research*.

[164] Herrmann J. L., Bruckheimer E., McDonnell T. J. (1996). Cell death signal transduction and Bcl-2 function. *Biochemical Society Transactions*.

[165] Kyriakis J. M., Avruch J. (1996). Sounding the alarm: protein kinase cascades activated by stress and inflammation. *Journal of Biological Chemistry*.

[166] Tibbles L. A., Woodgett J. R. (1999). The stress-activated protein kinase pathways. *Cellular and Molecular Life Sciences: CM*.

[167] Jun C. D., Pae H. O., Kwak H. J. (1999). Modulation of nitric oxide-induced apoptotic death of HL-60 cells by protein kinase C and protein kinase A through mitogen-activated protein kinases and CPP32-like protease pathways. *Cellular Immunology*.

[168] Palmer H. J., Paulson K. E. (2009). Reactive oxygen species and antioxidants in signal transduction and gene expression. *Nutrition Reviews*.

[169] Zhuang S., Demirs J. T., Kochevar I. E. (2000). P38 mitogen-activated protein kinase mediates bid cleavage, mitochondrial dysfunction, and caspase-3 activation during apoptosis induced by singlet oxygen but not by hydrogen peroxide. *Journal of Biological Chemistry*.

[170] Matsura T., Kai M., Fujii Y., Ito H., Yamada K. (1999). Hydrogen peroxide-induced apoptosis in HL-60 cells requires caspase-3 activation. *Free Radical Research*.

[171] Zhuang S., Lynch M. C., Kochevar I. E. (1998). Activation of protein kinase C is required for protection of cells against apoptosis induced by singlet oxygen. *FEBS Letters*.

[172] Zhuang S., Lynch M. C., Kochevar I. E. (1999). Caspase-8 mediates caspase-3 activation and cytochrome c release during singlet oxygen-induced apoptosis of HL-60 cells. *Experimental Cell Research*.

[173] Farley N., Pedraza-Alva G., Serrano-Gomez D. (2006). p38 mitogen-activated protein kinase mediates the fas-induced mitochondrial death pathway in CD8+ T cells. *Molecular and Cellular Biology*.

[174] Ono K., Han J. (2000). The p38 signal transduction pathway: activation and function. *Cellular Signalling*.

[175] Kharbanda S., Saxena S., Yoshida K. (2000). Translocation of SAPK/JNK to mitochondria and interaction with Bcl-x(L) in response to DNA damage. *Journal of Biological Chemistry*.

[176] Maundrell K., Antonsson B., Magnenat E. (1997). Bcl-2 undergoes phosphorylation by c-jun N-terminal kinase/stress-activated protein kinases in the presence of the constitutively active GTP-binding protein rac1. *Journal of Biological Chemistry*.

[177] Yamamoto K., Ichijo H., Korsmeyer S. J. (1999). BCL-2 is phosphorylated and inactivated by an ASK1/Jun N-terminal protein kinase pathway normally activated at G(2)/M. *Molecular and Cellular Biology*.

[178] Wang Y., Xia C., Lun Z., Lv Y., Chen W., Li T. (2018). Crosstalk between p38 MAPK and caspase-9 regulates mitochondria-mediated apoptosis induced by tetra-*α*-(4-carboxyphenoxy) phthalocyanine zinc photodynamic therapy in LoVo cells. *Oncology Reports*.

[179] Li P., Nijhawan D., Budihardjo I. (1997). Cytochrome c and dATP-dependent formation of apaf-1/caspase-9 complex initiates an apoptotic protease cascade. *Cell*.

[180] Chandra D., Choy G., Deng X., Bhatia B., Daniel P., Tang D. G. (2004). Association of active caspase 8 with the mitochondrial membrane during apoptosis: potential roles in cleaving BAP31 and caspase 3 and mediating mitochondrion-endoplasmic reticulum cross talk in etoposide-induced cell death. *Molecular and Cellular Biology*.

[181] Kuwana T., Smith J. J., Muzio M., Dixit V., Newmeyer D. D., Kornbluth S. (1998). Apoptosis induction by caspase-8 is amplified through the mitochondrial release of cytochrome c. *Journal of Biological Chemistry*.

[182] Kantari C., Walczak H. (2011). Caspase-8 and Bid: caught in the act between death receptors and mitochondria. *Biochimica et Biophysica Acta (BBA)—Molecular Cell Research*.

[183] Li H., Zhu H., Xu C. J., Yuan J. (1998). Cleavage of BID by caspase 8 mediates the mitochondrial damage in the fas pathway of apoptosis. *Cell*.

[184] Luo X., Budihardjo I., Zou H., Slaughter C., Wang X. (1998). Bid, a Bcl2 interacting protein, mediates cytochrome c release from mitochondria in response to activation of cell surface death receptors. *Cell*.

